# A Case of Cystic Dysplasia of the Rete Testis in a 17-Months-Old Boy

**DOI:** 10.1155/2011/389857

**Published:** 2011-07-03

**Authors:** Anna Poupalou, George Spyridis, Marina Vakaki, Panagiota Giamarelou, George Petousis, Pantelis Nikolaidis

**Affiliations:** ^1^Department of Pediatric Surgery, Hôpital Necker Enfants Malades, 149 Rue de Sévres, Paris, France; ^2^First Department of Pediatric Surgery, Panagiotis and Aglaia Kyriakou Children's Hospital, Athens, Greece; ^3^Department of Radiology, Panagiotis and Aglaia Kyriakou Children's Hospital, Thivwn-Levadias 1-3, Athens, Greece; ^4^Department of Pathology, Panagiotis and Aglaia Kyriakou Children's Hospital, Thivwn-Levadias 1-3, Athens, Greece

## Abstract

Cystic dysplasia of the testis (CDT) is a benign, congenital malformation of the testis and a rare cause of painless scrotal swelling in children, mimicking testicular cancer. It is commonly unilateral, often associated with ipsilateral wolffian duct and ureteral abnormalities. Cystic dysplasia of the rete testis (CDT) represents a diagnostic challenge made easier if age, precise localisation, typical ultrasonographic features, the presence or absence of associated genitourinary malformations, as well as tumor markers are considered. The definite treatment of such a benign lesion is testis-sparing surgery, however in most cases watch and wait strategy can be recommended. We present a case of cystic dysplasia of the testis in a 17-month-old boy with right multicystic dysplastic kidney, epididymal cyst, history of vesicoureteral reflux (VUR), as well as of solitary umbilical artery. We performed epididymidal cyst enucleation and right testicular biopsy.

## 1. Introduction

Cystic dysplasia is an unusual benign congenital lesion of the rete testis often associated with ipsilateral renal and ureteral abnormalities. Compression of the surrounding parenchyma generally occurs and may result in testicular atrophy. We present a case of cystic dysplasia of the testis in a 17-month-old boy.

## 2. Case Report

**Figure 1:**
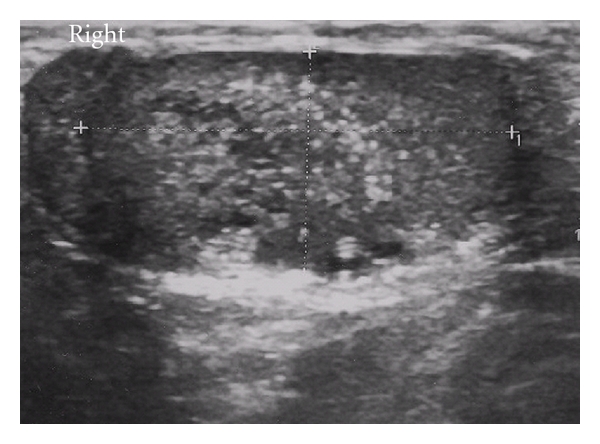
Scrotal ultrasound showing multiple small cystic lesions which comprise about 90% of the right testicular volume and are compressing the normal parenchyma to the periphery.

**Figure 2:**
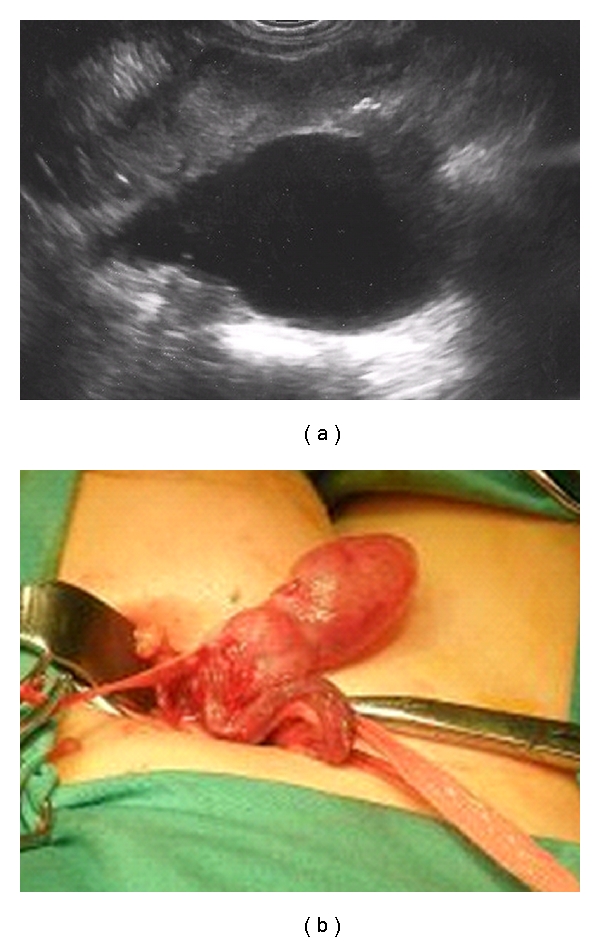
(a) Ultrasonic and (b) intraoperative images: the paratesticular mass, found to be an epididymal cyst was excised.

A 17-month-old white boy was admitted to the hospital with painless right hemiscrotal enlargement. The patient had a prior medical history of right multicystic dysplastic, ectopic, non functional on DMSA, kidney (MCDK) followed since gestation, a left VUR, and solitary umbilical artery at birth. On physical examination a paratesticular, possibly cystic mass, was palpated and the testis was found to be enlarged and nontender. The left testicle felt normal. Preoperative ultrasound showed right testicular enlargement (1.95 × 1.01 cm) compared to the left (1.50 × 0.62 cm). The multicystic lesion comprised approximately 90% of the testicular volume and was compressing the normal parenchyma to the periphery (Figure 1). Cystic dysplasia of the rete testis was included in the differential diagnosis. The ultrasonography also demonstrated the paratesticular cystic mass as well as the right multicystic dysplastic ectopic kidney with compensatory hypertrophy of the controlateral kidney (Figure 2(a)). Upon admission, lactate dehydrogenase (LDH), human chorionic gonadotropin (HCG), and alpha-fetoprotein (AFP) were drawn that returned normal. 

Surgical operation was carried out through an inguinal incision to remove the paratesticular mass. Given the opportunity the testis was inspected and was noted to be enlarged, whitish and rubbery on palpation. The decision was made to perform a wedge biopsy. The paratesticular mass was found to be a simple epididymal cyst that was enucleated intact (Figure 2(b)).

**Figure 3:**
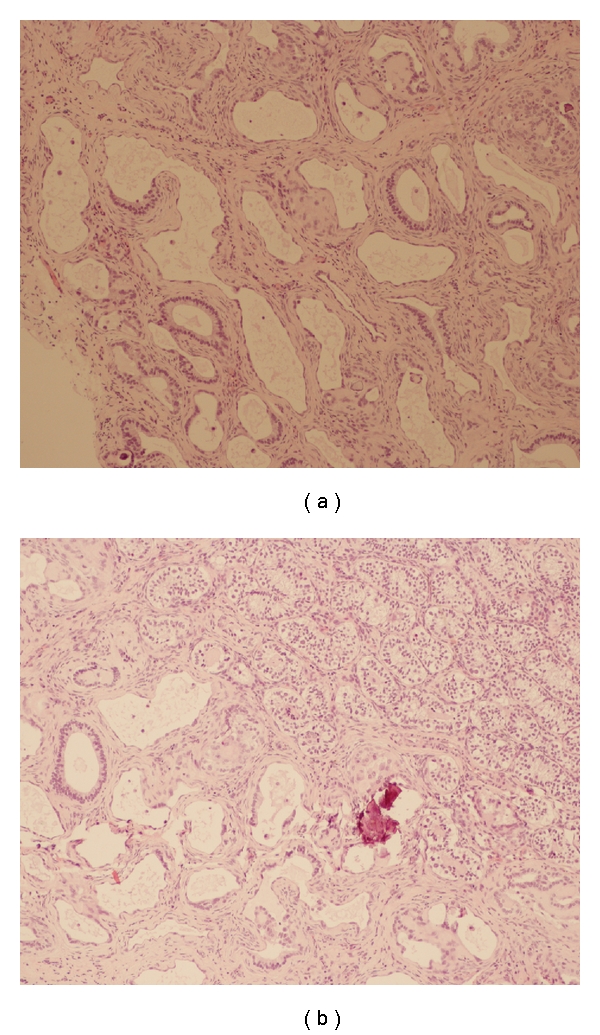
Histological examination showing (a) multiple, anastomosing, irregular cystic spaces of varying sizes and shapes, (b) pushing normal parenchyma toward periphery.

Histological examination showed multiple, anastomosing, irregular cystic spaces of varying sizes and shapes predominantly located in the mediastinum of the testis, consistent with cystic dysplasia of the rete testis (Figures 3(a) and 3(b)).

## 3. Discussion

First described in 1973, CDT is a rare congenital monolateral lesion resulting from abnormal development of the rete testis [1]. It is a benign tumefaction with a high incidence of associated ipsilateral renal agenesis [2]. Other associated urinary anomalies include multicystic dysplasia of the kidney, hydroureteronephrosis, vesicoureteral reflux, megaureter, and ureteral duplication [3]. Histologically, CDT is characterized by anastomosing cystic spaces lined by cuboidal or flat epithelium and separated by fibrous stroma. Immunochemical and ultrastructural studies have shown that the lining epithelium of CDT expresses keratin and vimentin and have concluded that it is similar to that of normal rete testis epithelium [4, 5]. 

CDT likely has an embryologic pathogenesis thought to arise during the fifth week of gestation. Normal testis development requires the interaction of the epigenitalis of the mesonephros, a Wolffian duct structure, and the rete tubules [6]. CDT is likely the consequence of such a disruption between the Wolffian duct and the rete tubules of the testis [5]. This failure of connection has been proposed as the possible mechanism leading to degeneration of the mediastinum testis into small cysts with ductal dilatation [7]. Similarly, normal renal development requires induction of the metanephric blastema by the ureteral bud, another Wolffian duct derivative. Disruptions in these interactions lead to anomalies such as renal agenesis and MCDK. The association between CDT and these renal abnormalities suggests a defect in the Wolffian duct at both the cephalic and caudal ends [5, 6]. 

This extremely rare cause of scrotal swelling may appear on physical examination as a painless transilluminating testicular mass [8]. Pain is very rarely seen, as the scrotum enlarges [6, 7]. One case which presented as an abdominal mass in a newborn together with multiple congenital anomalies has also been described [9].

Sonography is recognized as the imaging method of choice for the evaluation of the nature of testicular abnormalities as well as for the followup of untreated forms [1, 10, 11]. In CTD, the sonographic examination of the testis reveals multiple small cysts located in the mediastinum, whereas the surrounding testicular tissue appears to be normal, but compressed [4, 10]. The cysts range in size from microscopic to several millimetres and it may be diffused, involving the whole parenchyma or focal [11]. When the cysts are tiny, they may be sonographically demonstrated as echogenic foci mimicking testicular microlithiasis, a potential premalignant condition, though intraluminal calcification has been found in association to CDT [4, 11]. High frequency transducer is suggested to be of aid in the differential diagnosis of these entities [10]. 

Because of the frequent association of CDT with other ipsilateral genitourinary abnormalities, sonographic evaluation of the urinary tract should also be included in the diagnostic work up. Kogan proposes diagnostic criteria for CDT. They include the identification of cystic lesions on ultrasound, normal tumor markers, and evidence of associated ipsilateral mesonephric anomalies. If these criteria are met, he states that open biopsy is not necessary for diagnosis [6, 12]. 

Testicular tumor markers are an important tool in the evaluation of testicular tumors in children and adults [13]. High levels of tumor markers are usually associated with the diagnosis of malignant tumors or the recurrence of a treated malignancy. The preoperative evaluation of the levels of alphafetoprotein, human chorionic gonadotropin, and lactate dehydrogenase plays a significant role in patient selection for conservative management and followup. This is particularly attractive in prepubertal patients because many, if not most, tumors are benign in this population [13].

The differential diagnosis of cystic testicular lesions in children include epidermoid, dermoid, inflammatory, posttraumatic, or congenital testicular cysts [10]. Cystic degeneration after torsion, prepubertal teratoma, juvenile granulosa cell tumor, as well as testicular cystic lymphangioma, although rare in childhood, should also be considered [14]. Congenital cystic dysplasia of the rete testis should not be confused with ectasia of the rete testis, which is incidentally found in a large number of middle-aged and elderly men. This idiopathic condition is very often bilateral and is not usually associated with testicular enlargement or renal abnormalities [15].

On histologic examination of cystic dysplasia of the rete testis, the lesion consists of dilatation of the ducts and compression of the surrounding testicular tissue. The cystic spaces are lined with cuboidal cells and separated by fibrous septa. The cysts can be microscopic or measure up to several millimeters in size [7].

Historically, orchiectomy was recommended [1]. With improved understanding of the benign nature of this disorder, more conservative approaches (cyst excision, non-operative management) have come to the forefront [16]. High probability of spontaneous regression of the cystic dysplasia of the testis combined with its benign behavior makes observation the most reasonable initial approach [6, 17]. The primary benefit of this approach is the preservation of endocrine function and spermatogenic activity. However, the natural history of untreated CDT and its effect on normal testicular tissue are still unknown. Therefore, long-term followup is recommended [11]. Testis sparing surgery may be performed for significantly enlarged lesions or enlarging lesions under observation [12]. In our case, surgery was performed due to the presence of a cyst, of considerable size, of the epididymis.
